# Influence of the Quantity and Quality of Light on Photosynthetic Periodicity in Coral Endosymbiotic Algae

**DOI:** 10.1371/journal.pone.0043264

**Published:** 2012-08-17

**Authors:** Michal Sorek, Oren Levy

**Affiliations:** The Mina and Everard Goodman Faculty of Life Sciences, Bar-Ilan University, Ramat-Gan, Israel; Karlsruhe Institute of Technology, Germany

## Abstract

Symbiotic corals, which are benthic organisms intimately linked with their environment, have evolved many ways to deal with fluctuations in the local marine environment. One possible coping mechanism is the endogenous circadian clock, which is characterized as free running, maintaining a ∼24 h periodicity of circuits under constant stimuli or in the absence of external cues. The quantity and quality of light were found to be the most influential factors governing the endogenous clock for plants and algae. Unicellular dinoflagellate algae are among the best examples of organisms that exhibit circadian clocks using light as the dominant signal. This study is the first to examine the effects of light intensity and quality on the rhythmicity of photosynthesis in the symbiotic dinoflagellate Symbiodinium sp., both as a free-living organism and in symbiosis with the coral Stylophora pistillata. Oxygen production measurements in Symbiodinium cultures exhibited rhythmicity with a periodicity of approximately 24 h under constant high light (LL), whereas under medium and low light, the cycle time increased. Exposing Symbiodinium cultures and corals to spectral light revealed different effects of blue and red light on the photosynthetic rhythm, specifically shortening or increasing the cycle time respectively. These findings suggest that the photosynthetic rhythm is entrained by different light cues, which are wired to an endogenous circadian clock. Furthermore, we provide evidence that mRNA expression was higher under blue light for two potential cryptochrome genes and higher under red light for a phytochrome gene isolated from Symbiodinium. These results offer the first evidence of the impact of the intensity and quality of light on the photosynthetic rhythm in algal cells living freely or as part of a symbiotic association. Our results indicate the presence of a circadian oscillator in Symbiodinium governing the photosynthetic apparatus through a light-induced signaling pathway that has yet to be described.

## Introduction

The circadian clock is an endogenous mechanism that enables organisms to synchronize with the earth’s rotation with rhythmicity close to a 24-h cycle [1], [2]. This endogenous/inner clock perceives signals from the environment and makes adjustments to match the local time. The circadian clock’s true nature as an intrinsic clock is manifested in its ability to conserve a diel rhythm even in the absence of environmental signals, such as under conditions of constant light (LL) or darkness (DD) [3], [4]. In nature, the clock functions in harmony with the local surroundings and adjusts the organism’s based on certain environmental stimuli. Another important role of the endogenous circadian machinery is anticipation, which prepares the organism for predictable environmental changes. This function offers an evolutionary advantage for the organism’s survival and functioning [5]. In general, the endogenous clock system is comprised of three major components: the input pathway(s), the central oscillator mechanism and the output pathway(s). The mechanism underlying the central clock oscillator is based on one or more negative feedback loops, which are regulated by an array of genes and proteins that generate daily rhythms of 24 h and govern the expression of clock-controlled genes (CCG’s) in the output pathway. The central oscillator is synchronized by external signals, such as light and temperature, through the input pathways [4], [6], [7].

Light is considered to be a universal cue for synchronizing the clock. Dawn and dusk illumination constitute the most reliable cues for the day phase [8]. Light signals are perceived and transduced to the central oscillator via photoreceptors [9]. Two clusters of photoreceptors are responsible for mediating light signals to the plant oscillator: phytochromes (PHYs) and cryptochromes (CRYs), which perceive red and blue light, respectively [10], [11]. Plant cryptochromes are flavoproteins (FAD) with a structural similarity to DNA photolyase that lack the photolyase repair mechanism for UV light-induced damage to cyclobutane pyrimidine dimers. Cryptochromes absorb light in the blue region and are found throughout the plant kingdom, including angiosperms, ferns, mosses and algae [12]–[17]. In *Arabidopsis*, two genes encoding cryptochromes showing a significant absorption peak in the blue light region (370 to 450 nm) have been isolated [18], [19]. A response to a portion of the green light spectrum (550 nm) was also observed [20]. Phytochromes are chromoprotein homodimers that contain one covalently linked linear tetrapyrrole chromosphere per molecule. These photoreceptor molecules absorb red and far-red light, which activates and inactivates these molecules, respectively [21]. In *Arabidopsis*, five phytochrome genes have been identified and isolated [22], [23]. An impressive example of a circadian rhythmicity is presented by the photosynthetic apparatus, which is light-dependent.

Photosynthesis is governed and regulated by photoreceptors and by the core circadian clock oscillator. This well-known mechanism is found in both plants [24] and algae [25], [26]. Changes in light quality (light spectra) and quantity (irradiance levels) can affect the central clock’s synchronization and periodicity functions and cause mismatches in the output processes. Different organisms have been found to respond differently to changes in light intensities. Certain organisms show behavior in which the periodicity becomes shorter, whereas the periodicity in others becomes longer [27]. Increasing light intensity often shortens the periodicity in diurnal organisms, including plants. Nocturnal organisms display the opposite pattern [28]. Another characteristic of light is its spectrum. Different wavelengths can synchronize diverse processes in a plant’s life cycle, influencing the time period and the clock phase. The involvement of an array of photoreceptors in synchronizing the clock indicates that the spectral composition is an important signal, which varies during daylight hours from dawn to dusk [29]. In higher plants, such as *Arabidopsis*, the light spectrum, primarily the blue and red wavelengths, is required for maintaining circadian clock synchronization. The phytochromes and cryptochromes are the primary photoreceptors and their signals are transduced to the main oscillator [4]. These photoreceptors also govern genes involved in the photosynthetic machinery, such as chlorophyll a/b-binding protein (CAB) [10], [11].

In the dinoflagellate *Gonyaulax polyedra,* two known light input pathways are linked with the circadian clock machinery. The first pathway responds to both blue and red light spectra, whereas the second only responds to a portion of the blue light spectrum [30]. Identical pathways were discovered in *Chlamydomonas* [31]. Spectral light effects on vegetative growth, oxygen evolution and bioluminescent outputs have been examined in relation to circadian clock periodicity in the red algae *Porphyra umbilicalis* and *Kappaphycus alvarezii*, as well as the dinoflagellate *Gonyaulax polyedra* [27], [29], [32], [33]. In each of these algae, an opposite response to blue light (short wavelength) compared to red light (long wavelength) was found. With increasing irradiance, the free running period decreased under blue light and increased under red light. In these algae, there is an obvious response to different light wavelengths with respect to the circadian rhythm. This finding implies that there are two kinds of photoreceptors in these algae. However, these photoreceptors have not yet been identified. The goal of this work was to examine the effects of light intensity and quality on the rhythmicity of photosynthesis in the dinoflagellate *Symbiodinium* sp. In this study, we performed a detailed comparative analysis of the photosynthetic time periodicity in cultured *Symbiodinium* algae (clade A) and in the symbiotic coral *Stylophora pistillata* (associated with *Symbiodinium* algae, clade A, C) by measuring oxygen evolution as an output process. These processes have not been investigated previously with respect to the intensity and quality of light (light spectra consisting of blue, green and red). In addition, we measured the changes in the mRNA expression of the putative cryptochrome (CRY1, CRY2) and phytochrome (PHY) genes during LD and LL conditions under red and blue light spectra.

## Materials and Methods

### Collection and Maintenance of Corals and *Symbiodinium* Cultures

The *Symbiodinium* cultures used in this research belonged to clade A (ccmp 2467). All cultures were grown in 2-L Fernbach flasks containing 1 L of half-strength medium (f/2) without silica. Illumination was provided by lateral fluorescent lamps under a 12∶12 h LD cycle. The cultures were maintained in a culture room with a controlled temperature set to 24°C for all indoor experiments. Irradiance was measured with a quantum sensor (LI-COR, Lincoln, NE), and measurements of 100 µmol quanta m^−2^ s^−1^ were obtained. SCUBA divers collected the *Stylophora pistillata* corals tested in this study from a depth of 5 m from in front of the Interuniversity Institute (IUI) for Marine Sciences, Eilat, Gulf of Aqaba, Red Sea, Israel. The Israeli Nature and National Parks Protection Authority approved the collection of corals for this study (permit No. 2011/38353). After collection, the corals were fragmented and placed in running seawater and then transferred to 900-L aquaria for a period of one week for acclimation. Later, the corals were transferred to the new Aquarium System Facility recently established by the Levy group at Bar-Ilan University, Israel. Coral fragments were acclimated for another week prior to the light intensity and spectra experiments.

#### Oxygen evolution monitoring

Oxygen evolution was monitored in cultured *Symbiodinium* with a 4-channel oxygen meter (OXY4) equipped with 4 optical oxygen sensors (OPTODs; PreSens, Germany) connected to 4 dipping probes (DP-PSts). The OPTODs continuously monitored the oxygen evolution at 5-min intervals. We used this system to characterize the oxygen-evolving rhythm in all of the experiments described in this paper. The influences of light intensities and light spectra on the periodicity of photosynthesis were tested on clade A. Prior to the experiments, the oxygen content in medium-only flasks (free of algae) was measured as a baseline to remove any potential artifacts due to drifting of the OXY-4 probe and the f/2 medium. The light intensities were reduced during the experiments from 100 to 75 or 50 µmol quanta m^−2^ s^−1^. The intensity levels were adjusted by measuring the light with a quantum sensor (LI-COR, Lincoln, NE). For each light intensity tested, the four cultures were exposed to an LD cycle followed by two constant cycles of LL. To achieve blue, green and red light spectra during the light spectra experiments, we used a Lumen Aqua LED lighting system (Ocean Lights©, USA). This unique LED lamp system enabled us to achieve the desired spectra and intensities of 50 and 25 µmol quanta m^−2^ s^−1^. The light spectrum quality was measured with an HR4000 High-Resolution Spectrometer (Ocean Optics). Oxygen production of *Symbiodinium* cultures were first monitored in cultures exposed to spectral light under LD conditions followed by two cycles of LL under the same spectral light conditions. *Stylophora pistillata* fragments were measured under different light spectra (*N = 4*, fragments for each light spectra tested) using the same methods employed for the *Symbiodinium* cultures. An OXY-4 probe was used, and we monitored the oxygen evolution during one cycle of LD followed by two cycles of LL. We used blue, red and green light at intensities of 50 µmol quanta m^−2^ s^−1^.

### qPCR of the CRY1, CRY2 and PHY Genes

#### *Symbiodinium* RNA extraction

*Symbiodinium* cultures belonging to clade A were grown at 24°C under different light spectra with a light intensity of 50 µmol quanta m^−2^ s^−1^ under LD cycles of 12∶12. The cultures were sampled at the following times: 06∶30 (90 min before light exposure), 08∶30 (30 min after light exposure began), 12∶00 (4 h after light exposure began), 16∶00 (8 h after light exposure began) and 20∶30 (30 min after light exposure ended). In addition, the cultures were exposed to LL conditions and sampled at the same times as in the LD experiment for each time point *N = 3*. For RNA extraction, 50 ml of *Symbiodinium* culture was collected by centrifugation (6,500 rpm for 5 min at 4°C). The cell pellets were then suspended in 500 µl of acidic phenol (Sigma) and 500 µl of NAES extraction buffer (50 mM sodium acetate buffer, 10 mM EDTA, and 1% SDS, pH 5.0) with 100 mg of glass beads (<106 µm average diameter; Sigma). The mixture was next vortexed for 1 min and immediately incubated for 5 min at 65°C. This process was repeated twice. Subsequently, the tubes were placed on ice for 10 min and subsequently centrifuged at 12,000 rpm for 7 min at 4°C. The upper phase was removed to a clean tube containing 250 µl of chloroform together with 250 µl of phenol, vortexed for 1 min and centrifuged at 12,000 rpm for 10 min at 4°C. The upper aqueous phase was later removed and transferred to a new tube containing 500 µl of chloroform, which was vortexed for 1 min and centrifuged at 12,000 rpm for 10 min at 4°C. After this stage, the upper phase was removed into a new tube containing 1 ml of ethanol (100%) and 120 µl of 8 M LiCl and precipitated overnight at −20°C. After centrifugation at 14,000 rpm for 10 min at 4°C, the flow-through was discarded. The RNA pellet obtained was washed twice with 1 ml of 75% ethanol and centrifuged at 14,000 rpm for 15 min. The flow-through was discarded, and the pellet was dried for 10 min. RNA was eluted via addition of 30 µl of RNase-free water and cleaned with RNeasy Mini columns (QIAGEN). RNA quantity and integrity were assessed with a NanoDrop ND-1000 spectrophotometer and an Agilent 2100 Bioanalyser, respectively. RNA with an integrity number (RIN) >9 was kept at −80°C for qPCR analysis. Using 1 µg of extracted total RNA, cDNA was synthesized using the Verso cDNA synthesis kit (Thermo scientific) following the kit’s protocol. Primers were designed for the qPCR assay using the Primer Quest software, Primer3 (v 0.4.0; source code available at http://fokker.wi.mit.edu/primer3/), for the CRY1, CRY2 and PHY genes based on EST data from KB8 *Symbiodinium* - clade A established by the Monica Medina group at the University of California, Merced (data available at http://medinalab.org/zoox ). The transcriptomes obtained were sequenced by the Joint Genome Institute (JGI), and the raw data were deposited in the Short Read Archive (SRA) at NCBI under the accession numbers SRX076710, SRX076709 and SRX076696. The primer sequences, melting temperatures and the amplicon product sizes used in these assays were as follows:

CRY1∶118 bp, Tm = 60°C

F-TCCGGTACGTTGTGTCCTTCGATT,

R-AGCCTCCGATTTCCAGAAGAGCA

CRY2∶143 bp, Tm = 60°C

F-TAAGGATCTGCCAGGATTGCCGTT,

R-AGTGCGGGAGTTTCAAAGGAGGA

PHY: 111 bp, Tm = 60°C

F-ATCGAGGAAAGCAATGTGCAACCC,

R-CGGAATTTGCCCACCGATGTTGAA

#### qPCR assays

The qPCR assays were performed using the Corbett RG6000 real-time detection system and the GoTaq qPCR Master Mix. Each 20-µl reaction contained 5 µl of GoTaq, 0.25 µl of 10 mM solutions of both forward and reverse primers, 1.5 µl of ddw and 3 µl of diluted cDNA (1∶200). The following thermal profile was used: 94°C for 7 min followed by 45 cycles of 94°C for 7 s, 60°C for 15 s and 72°C for 20 s. The runs were analyzed using the Rotor-Gene 6000 series software (v 1.7) and were further analyzed using Excel®-based tools that we designed. The fluorescence threshold was set at 0.02 for all runs. The CRY1, CRY2 and PHY genes were normalized against several housekeeping genes (HKG): Actin, Cyclophin and S-adenosyl-L-methionine synthetase. However, Cyclophin was chosen as the most robust and stable HKG for our experiments, identical to the findings of Rosic et al. [34]. The relative expression of each sample and gene was calculated using the 2^−ΔΔCT^ method; the corresponding real-time PCR efficiencies (E = 10 ^[–1/slope]^) for all genes tested was E = 1.9, calculated using the Rotor-Gene 6000 series software (v 1.7).

#### Data analysis

Oxygen data sampled on the time domain were transformed into the frequency domain via Fourier transformation. For this purpose, we coded an application in the Python environment using the ‘scipy’ library function ‘fft’. From the output of the application, we extracted the dominant frequencies that characterized the examined rhythm. For statistical analysis, paired t-tests and one-way ANOVA followed by Tukey’s HSD were used to assess the differences between the experimental treatments regarding oxygen periodicity and gene expression under different light conditions. All statistical analyses were conducted using SPSS 20.0 (IBM, USA), and the results were considered to be statistically significant at *P*<0.05.

## Results

### Rhythm of Free-running Oxygen Evolution Under Different Light Intensities

**Figure 1:**
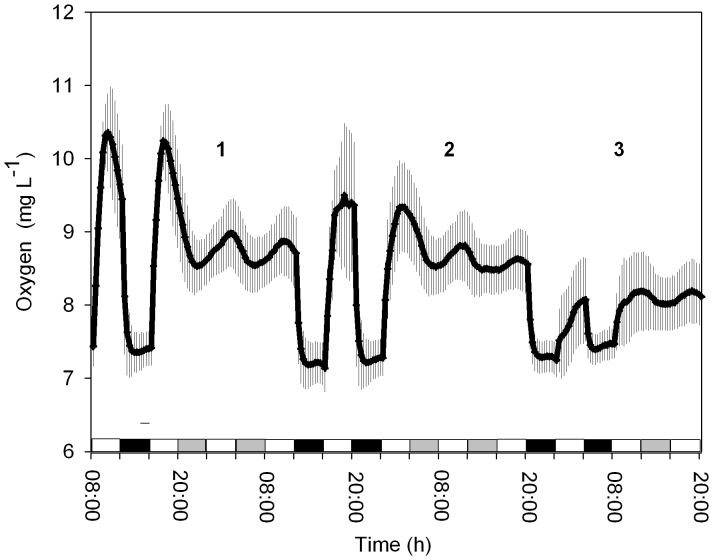
Oxygen evolution during the LD cycle at 3 different light intensities followed by LL cycles. 1) 100 µmol quanta m^−2^ s^−1^, 2) 75 µmol quanta m^−2^ s^−1^ and 3) 40 µmol quanta m^−2^ s^−1^. White bars indicate the light period, black bars the dark period and grey bars subjective darkness (Each time point *N = 4,* ±S.D.).

The temporal oxygen production from the *Symbiodinium* cultures (clade A) under light intensities of 100, 75 and 50 µmol quanta m^−2^ s^−1^ was analyzed. The periodicity of the free-running cycles was evaluated via a Fourier transform and converted into a time period. Under LD conditions (full spectrum, 400–700 nm), the rhythmicity of photosynthesis achieved a 24-h cycle for each light intensity tested (Figure 1). Under a high light intensity (100 µmol quanta m^−2^ s^−1^), the cycle was 24±0.3 h; for the medium light intensity (75 µmol quanta m^−2^ s^−1^), the cycle was 24±0.12 h; and for the low, dim light intensity (50 µmol quanta m^−2^ s^−1^), the rhythm was 24.05±0.4 h (*N = 4*, for each light intensity tested). In subsequent continuous light (LL conditions) experiments, persistent free running with a period close to 24 h (23.6±0.35 h, *N = 4*) was observed only under the high light condition (100 µmol quanta m^−2^ s^−1^ culture growing conditions). An increase in the free-running oxygen production period was observed in the medium (24.38±0.14 h, *N = 4*) and dim (25.3±0.17 h, *N = 4*) light intensities (75 and 50 µmol quanta m^−2^ s^−1^, respectively) (Figure 1), and they were found to be significantly different from those under the high light intensity tested (one-way ANOVA followed by Tukey’s HSD, *P*<0.05). These results under full light spectra indicate the existence of a circadian clock governing photosynthesis in *Symbiodinium.*

### Rhythm of Free-running Oxygen Evolution Under Different Light Qualities

**Figure 2:**
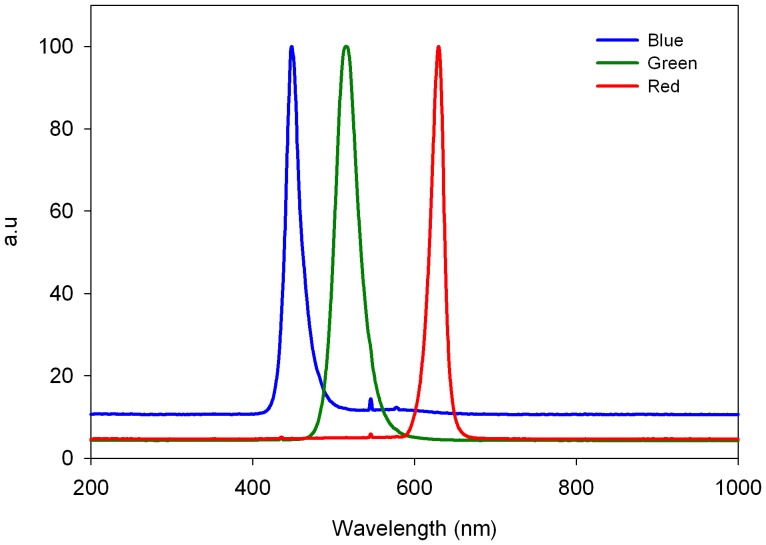
Spectral distributions of light fields obtained using an epro Lumen lamp and measured with an HR4000 High-Resolution Spectrometer (Ocean Optics) for red, blue and green spectra.

**Figure 3:**
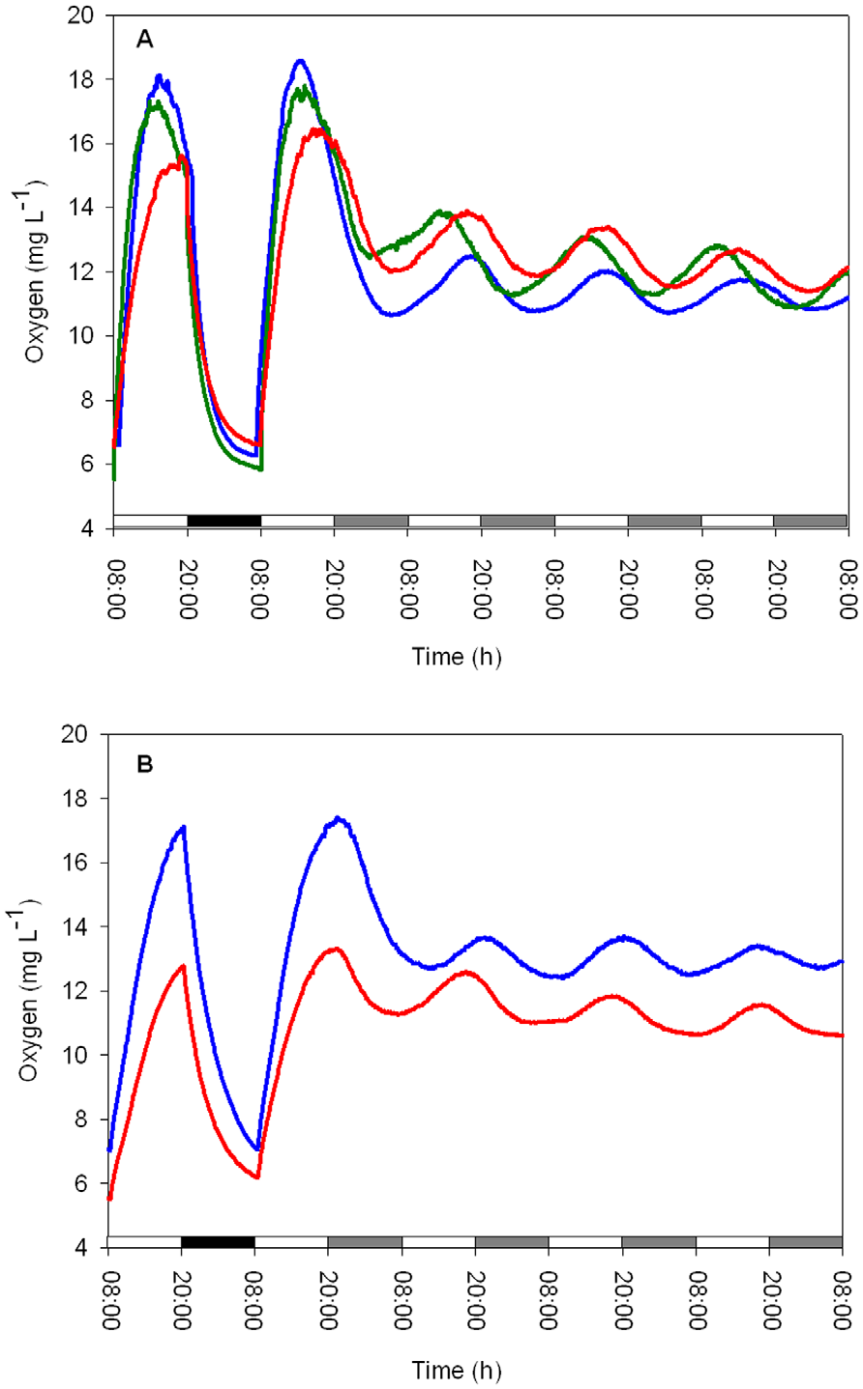
Oxygen evolution of cultured algae during an LD cycle (A) followed by 4 LL cycles under red, blue or green spectral light with a uniform light intensity of 50 µmol quanta m^−2^ s^−1^. B) The same setup with a lower light intensity of 25 µmol quanta m^−2^ s^−1^ (Each time point *N = 4*).

**Figure 4:**
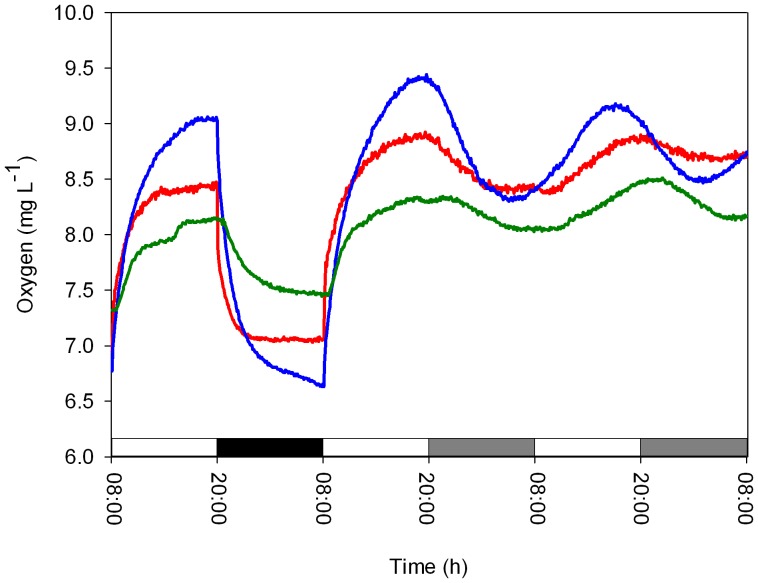
Oxygen evolution from *Stylophora pistillata* corals during an LD cycle followed by 4 LL cycles under red, blue or green spectral light with a uniform light intensity of 50 µmol quanta m^−2^ s^−1^ (Each time point *N = 4*).

To determine whether the quality of the light had an effect on the circadian oscillation of oxygen evolution, experiments were performed using blue/green or red lights (Figure 2) at two irradiance levels (50 and 25 µmol quanta m^−2^ s^−1^) under LD and LL cycles. During the LD cycle, cultures exposed to blue, green and red lights (irradiance level, 50 µmol quanta m^−2^ s^−1^) showed diel rhythms of oxygen production with approximately 24-h cycles (blue: 24±0.45 h, green: 24±0.16 h and red: 24±0.21 h, *N = 4* per treatment, Figure 3A), with no significant difference between treatments (one-way ANOVA, *P* = 0.65). Substantial differences between the three light spectra tested were observed in the free-running period, τ, calculated from the Fourier transform during the LL treatments (irradiance level, 50 µmol quanta m^−2^ s^−1^). Periodicities of 23.5±0.17 h and 23.5±0.11 h were detected for the blue and red light spectra, respectively, which were significantly different from the short green light period of 22.7±0.21 h (one-way ANOVA followed by Tukey’s HSD, *P*<0.01, *N = 4* per treatment, Figure 3A). Deviation in the amplitude of oxygen production was observed after the first day of continuous light under all three light spectra tested. This result is presumably related to the ability of certain pigments to absorb specific wavelengths of light for photosynthesis. The alteration of the temporal periodicity of the free run may be related to the entrainment cued by an array of photoreceptors that signal to the core oscillator. The *Symbiodinium* cultures tested under LD dim (25 µmol quanta m^−2^ s^−1^) blue and red light spectra exhibited a diel period of 24±0.02 h (*N = 4*) (Figure 3B), while the free run revealed a significant difference between the two light spectra. Cultures under constant blue light conditions maintained a rhythmicity of 24.4±0.1 h, whereas under a red light cycle, the rhythmicity was significantly shortened to 22.7±0.4 h (paired t*-*test, *P*<0.01). Comparison of the τ values under the blue light cycles showed a longer cycle under dim blue light (24.4±0.1 h) compared to high blue light (23.5±0.17 h) (paired t*-*test, *P* = 0.03). The opposite trend was detected for the τ values between the high red light (23.5±0.11 h) compared with dim red light (22.7±0.4 h) (paired t*-*test, *P* = 0.03) (Figure 3A, B). The temporal oxygen evolution measured in *Symbiodinium* algae associated with the coral *Stylophora pistillata* showed patterns that were distinct from the photosynthetic patterns measured in cultured algae exposed to the three light spectra. There were differences in the free-running periodicity under the blue and red light regimes (50 µmol quanta m^−2^ s^−1^) when switching from LD to LL. Under blue light, the corals switched from a diel cycle of 24.1±0.11 h in LD to a shorter *τ* cycle of 23.8±0.14 h under LL (paired t*-*test, *P*<0.05, *N = 4*), whereas under red light treatment (with the same intensity), a diel cycle of 24.6±0.12 h under LD conditions and a longer free cycle of 25.1±0.19 h of oxygen production (paired t*-*test, *P*<0.05, *N = 4*) were observed (Figure 4). For green light, the time period under LD calculated by the Fourier transform was 24.4±0.21 h. Under LL, the free run was shortened to 22.76±0.15 h (paired t*-*test, *P*<0.01, *N = 4*). In *Symbiodinium* embedded in the coral *Stylophora pistillata,* temporal oxygen production showed similar responses of shortening the cycle under LL conditions when measured under the higher light intensity (50 µmol quanta m^−2^ s^−1^) for the three light spectra. All of the results for oxygen evolution periodicity under spectral light analyzed using Fourier transform are summarized in Table 1.

**Table 1:** The diel periods length of oxygen evolution of cultured algae and *Stylophora pistillata* corals under different light spectra and intensities calculated using Fourier transform.

Organism	Spectral light	Intensity(µmol quanta m^−2^s^−1^)	LD (h)	LL (h)
*Symbiodinium*	Blue	50	24.5±0.45	23.5±0.17
*Symbiodinium*	Blue	25	24±0.02	24.4±0.1
*Symbiodinium*	Red	50	24±0.21	23.5±0.11
*Symbiodinium*	Red	25	24±0.02	22.7±0.4
*Symbiodinium*	Green	50	24±0.16	22.7±0.21
*Stylophora pistillata*	Blue	50	24.1±0.11	23.8±0.14
*Stylophora pistillata*	Red	50	24.6±0.12	25.1±0.19
*Stylophora pistillata*	Green	50	24.4±0.21	22.76±0.15

### Cryptochrome and Phytochrome Genes Expression

To investigate further the potential involvement of photoreceptors in cueing an array of physiological, metabolic and behavioral circadian cycles in *Symbiodinium* algae (e.g., oxygen production), the temporal gene expression of two potential candidate CRY genes and one PHY gene were tested in relation to light spectra.

**Figure 5:**
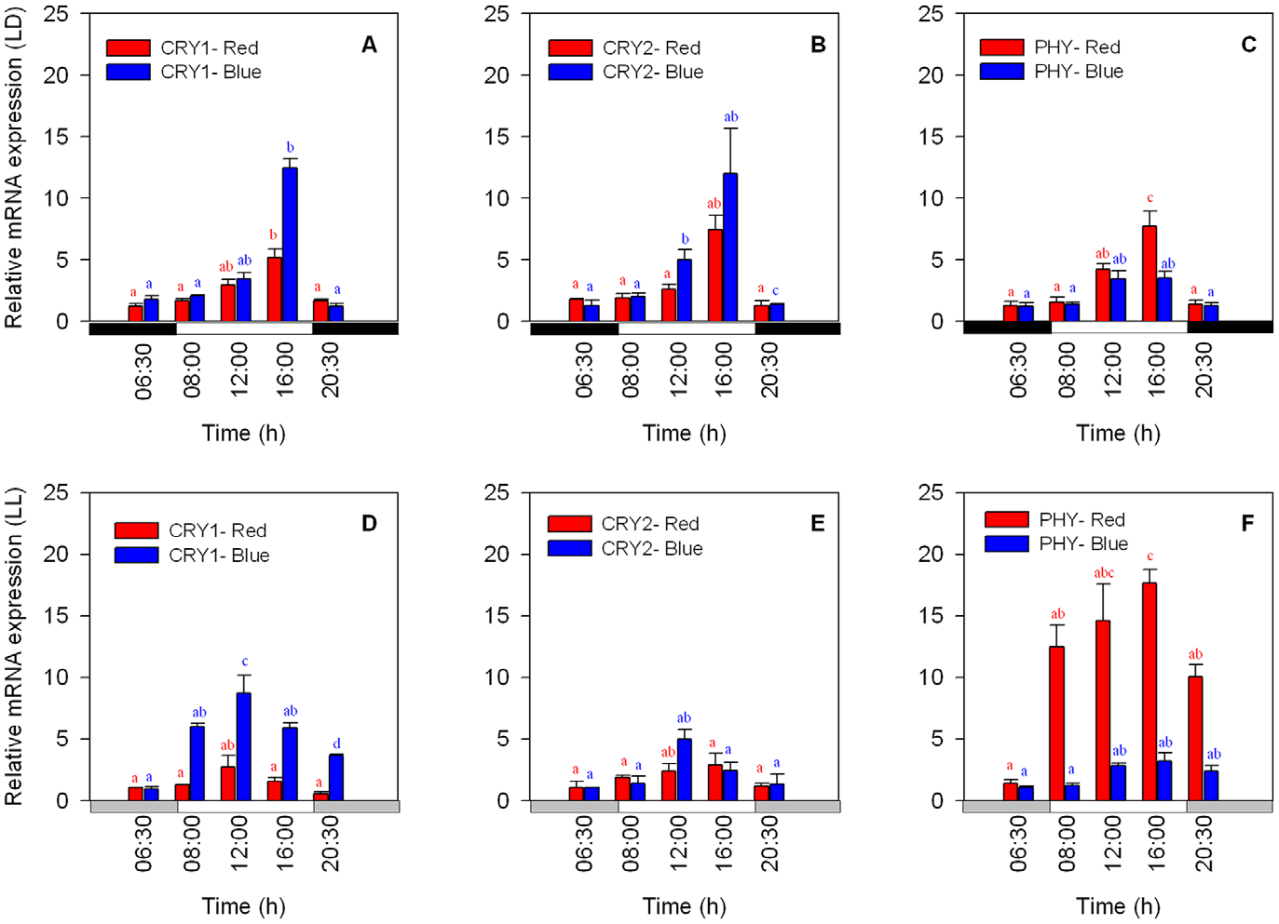
Expression of mRNA as determined by qPCR analysis of the putative CRY1, CRY2 and PHY genes. A–C) LD cycle, 12∶12 h (8∶00–20∶00); D–F) LL cycle. White bars indicate the light period, black bars the dark period and grey bars subjective darkness (Each time point *N = 3,* ±S.D.). Significantly different groups from one-way ANOVA followed by Tukey’s HSD test are shown with different letters (*a*, *b*, ab, *c* and *d*) (*P*<0.05).

The mRNA expression of the putative CRY1, CRY2 and PHY genes showed a daily peak during the LD treatment. The sequential gene expression was similar for all three genes. Gene expression began to increase with the first light applied (30 min after lights were turned on) and reached maximum expression at 16∶00 (8 h after the lights were turned on). This was followed by a decline in expression at 20∶30 (30 min after the lights were turned off) (Figure 5 A–C). Blue light appears to have stimulated the expression of CRY1 and CRY2 in the same manner, with similar expression levels being observed and maximum expression peaking at 16∶00 in both cases (one-way ANOVA followed by Tukey’s HSD, *P*<0.01, *N = 3* per time point) (Figure 5 A, B). The PHY gene also exhibited a peak at 16∶00, but showed much lower expression levels under blue light. Under red light, all 3 genes showed rhythmicity resembling that under blue light, but the expression level of PHY was higher in comparison with the CRY genes, particularly when peak expression was observed at 16∶00 (one-way ANOVA followed by Tukey’s HSD, *P*<0.05, *N = 3* per treatment) (Figure 5 C). In *Symbiodinium* algae sampled under constant blue and red light conditions (LL), the expression of CRY1 under both types of spectral light showed an earlier peak at 12∶00 (one-way ANOVA followed by Tukey’s HSD, *P*<0.05, *N = 3* per treatment). The expression levels were higher under blue spectrum light, but they were lower in comparison to the expression levels under the LD treatments (Figure 5 D). The same temporal pattern was observed for CRY2, with an earlier expression peak occurring at 12∶00 (one-way ANOVA followed by Tukey’s HSD, *P*<0.01, *N = 3* per treatment). Lower values were observed under the LL conditions, with higher expression being detected under the blue spectra vs. the red spectra (Figure 5 E). Generally, the CRY expression levels were more than twofold higher under exposure to blue light for both LD and LL conditions compared to the values obtained under red light (Figure 5 A–B, D–E). The temporal expression levels of PHY under LD conditions were similar to the CRY expression patterns, showing a significant peak at 16∶00, with higher expression levels detected under the red light conditions in comparison with the blue light spectra (Figure 5 C) (one-way ANOVA followed by Tukey’s HSD, *P*<0.01, *N = 3* per treatment). PHY expression under constant red or blue light conditions exhibited an opposite trend to that of CRY, with significantly higher (almost three-fold higher) gene expression being detected under constant red light in comparison to the LD conditions (see Figure 5 C, F) (one-way ANOVA, *P*<0.01, *N = 3* per treatment). PHY mRNA expression under blue light was not influenced by the constant conditions, and similar levels of expression were maintained under both conditions. In contrast to the CRY genes, PHY did not show a time phase shift under LL conditions.

## Discussion

Our previous data on *Symbiodinium* photosynthesis (either associated with a coral/host or as unicellular free algae) show a circadian pattern (see Figure 1; Sorek et al. 2012, Unpublished). Elucidating the effect of light spectra on the periodicity of *Symbiodinium* oxygen production as a downstream output of the circadian machinery is not a trivial task. The current study obtains these data by evaluating the photosynthetic rhythm in cultured algae and in the symbiotic coral *Stylophora pistillata.* The study tested three light spectra (blue, green and red) at two irradiance levels. The temporal oxygen production results presented herein support the theory that the circadian machinery in *Symbiodinium* governs its photosynthetic apparatus. Daily oscillations were detected during the LD cycles at each of the light intensities tested (Figure 1). Under LL conditions, free running under treatment with 100 µmol quanta m^−2^ s^−1^ yielded an approximately 24-h rhythm. However, a longer periodicity close to 25.5 h was observed when the cultures were switched to lower light intensities of 75 and 50 µmol quanta m^−2^ s^−1^. Nevertheless, this longer free-running period still meets the criteria defined for circadian rhythms, which range between 19–28 h [35] (Figure 1). The shortening of cycles in response to decreasing light intensities has been reported for many photosynthetic organisms [36]. This also occurred in the *Symbiodinium* cultures tested in this work. The preserved rhythm under constant light at different irradiance levels illustrates the control exerted by the endogenous clock in this organism.

The calculated free-running cycles of oxygen evolution under spectral light (blue, green and red) revealed another layer of control of the endogenous clock in *Symbiodinium,* indicating entrainment due to light quality (Figure 3 A, B). Analysis using a Fourier transform demonstrated an ability to entrain the algae and maintain diel photosynthesis with a 24-h period when the algae were treated with the three light spectra tested during LD; under LL conditions, the rhythm under green light showed shorter cycles compared to the rhythms under blue and red light (Figure 3A). The endogenous circadian system of *Symbiodinium* managed to preserve the rhythm under the blue and red spectra (approximately 23.5 h), but was less efficient under green light, where the cycle tended to occur in a significantly shorter period (22.7 h) (Figure 3A). The ability of *Symbiodinium* to preserve a periodicity of approximately 24 h in the blue and red light portions of the spectrum implies the existence of cryptochromes and phytochromes and demonstrates that they play a role in entraining the core circadian clock machinery [4].

In the green spectrum, the pace was altered, but the algae preserved rhythmic photosynthesis. This result may be related to the fact that cryptochrome molecules are also known to exhibit absorptivity in the green light region [20], or “leaking” of blue light may have caused contamination when the algae were illuminated with the green light (see Figure 2). In the natural environment, the spectral light composition fluctuates. Twilight hours are characterized by shorter wavelengths (<500 nm) compared to the midday hours (500–650 nm). This fluctuation allows organisms to double the amount of information they receive by absorbing both blue/green and red light to measure time more accurately and obtain input for their circadian clock [35]. In dinoflagellates of the genus *Gonyaulax* [37], two types of light inputs, namely, blue and red, were observed to regulate the circadian clock machinery. These pathways are apparently responsible for synchronizing two separate endogenous clocks, which, in turn, are responsible for different output responses, including the bioluminescence rhythm and vertical migration in the water column, as well as phototactic orientation and photosynthesis [27], [38], [39]. The oxygen measurements performed in *Symbiodinium* cultures under low blue and red light intensities showed an increase in the free-running cycle length under the blue spectrum and a decrease in the period under the red spectrum (Figure 3 B).

Similar results have been reported in previous works [27], [30], [32], [33]. The above findings imply that robust spectral light signaling to the *Symbiodinium* circadian core oscillator takes place. However, our inability to produce specific mutants deficient in CRYs and PHY in *Symbiodinium* algae means that our findings are currently only based on physiological and gene expression patterns. We assume that the same photoreceptor components act in an input pathway in *Symbiodinium* in a similar fashion as reported for the excitation of cryptochrome photoreceptors in *Chlamydomonas reinhardtii* [17] and, more recently, in the algae *Ostreococcus* [40]. In contrast to the free-running cycles observed in the unicellular *Symbiodinium* cultures, the temporal photosynthesis pattern in the coral *Stylophora pistillata* exhibited different behavior when this species was treated with the same intensity and quality of light. Blue light caused a shortening of the cycle (23.8 h), while red light increased its periodicity (25.1 h). Green light shortened the cycle in the same manner as in the cultured algae. As *Symbiodinium* is “hospite” in the coral endoderm tissue layer, it is likely there is a shift in the spectral light reaching the algae embedded within the host. Corals are known to contain a diverse array of host pigments, many of which are critical to their light absorption capabilities, such as the Green Fluorescent Proteins (GFPs) [41], [42]. D’Angelo et al. [41] found that an increase in coral pigmentation was dependent on the spectral quality of light, with blue light levels being the key driver of pigment concentrations. This additional pigmentation in the coral may also be the cause of the differences in the free cycles found between the free *Symbiodinium* cultures and the symbiotic corals when exposed to constant spectral light conditions. Another possibility may be associated with the way that different input pathways respond in the symbiotic form and the fact that mainly blue and green light are available under water.

The temporal expression patterns of the potential photoreceptor genes CRY and PHY under spectral light treatment showed higher expression levels correlated with light hours, with maxima being reached a few hours after the lights were turned on (Figure 5). Lower expression was closely related to the period of darkness. This pattern was maintained under LD and LL conditions for both the blue and red spectra. However, some differences were observed during the constant light conditions, such as maximum expression occurring earlier (12∶00) and lower expression levels being detected for the CRY genes (Figure 5 A–B, D–E). Blue light caused approximately twofold higher expression levels of CRY1 and CRY2 under both treatments (LD and LL) in comparison with the red spectrum. Cryptochromes do not respond to red light and usually show no absorbance in the red light spectrum [20]. However, CRY gene promoters in *Arabidopsis* showed low expression levels under DD (and presented a circadian pattern for CRY1) [9]. Therefore, the expression of CRYs under red light may be considered similar to that under DD, assuming that CRYs are “blind” to the red spectrum. For the PHY gene, the opposite behavior was observed when the effects of LD and LL conditions were observed under red light. The mRNA expression levels detected under LL and red light were found to be much higher (by more than threefold) than under LD, but under blue light, the PHY mRNA levels did not show any difference between LD and LL and also remained low compared to those observed under red light (Figure 5C, F). The low expression levels of PHY under the blue spectrum can be explained by the fact that phytochromes in *Arabidopsis* also respond to the blue spectrum [21] and that the circadian oscillation of PHY under blue light may be the result of CRY1 and CRY2 acting as signal transduction components downstream of the phytochromes [10]. Photoreceptors in plants mediate the light input to the central clock, and both cryptochromes and phytochromes exhibit circadian gene expression under constant light conditions [18], [43], [44].

Studies on *Arabidopsis* PHY genes have revealed circadian rhythmicity based on mRNA accumulation and GUS reporter data [22], [45], [46]. Similar findings have been reported for CRY promoters fused with luciferase, revealing robust circadian oscillations under LL. This result was also observed under constant darkness for CRY1 [9]. The fact that photoreceptor genes in *Symbiodinium* exhibit similar temporal expression under LD and LL with both types of spectral light may imply the existence of more than one regulatory loop [9]. The central clock regulates photoreceptor mRNA levels as output processes in addition to the known role of photoreceptors in plants as an input pathway that synchronizes the clock. The expression patterns of the potential photoreceptor candidates in this study illustrate that *Symbiodinium* possess an endogenous circadian oscillator that is most likely cued by different light spectra. At this point of the study, we cannot exclude the possibility that PHY and CRY may be part of the circadian clock machinery. The presence of these potential photoreceptors probably mediates photosynthetic rhythmicity, which is controlled by the main circadian pacemaker; this effect could help in maintaining coral-*Symbiodinium* photosynthetic rhythmicity in the marine environment, where the underwater light decreases exponentially with depth, roughly following the Beer-Lambert law [47]. The exponential reduction in aquatic light intensity with depth is partly attributed to the absorbent properties of the water itself [48]. Light attenuation is not uniform over all wavelengths, and the water column behaves similar to a monochromator, narrowing the spectrum of the most penetrating light to a relatively narrow waveband [49], [50]. In the clear, tropical waters surrounding symbiotic coral reefs, light extinction in the violet and blue wavelengths is minimal, while its attenuation is higher at longer wavelengths. Therefore, the CRY and PHY photoreceptors of *Symbiodinium* may react differently in signaling the circadian oscillator in corals living in shallow water compared to corals living in deeper water that mainly receive crystal blue light. Another important issue is that in our study, *Symbiodinium* associated with the coral *Stylophora pistillata* are being inherited from the parent colony in a process known as vertical transmission. In contrast, other coral species acquire their *Symbiodinium* from their surroundings, which is a phenomenon known as horizontal transmission. How adaptable and synchronized is the clock of the symbiotic algae under both strategies is a key evolutionary question which we hope to answer in the future. The signaling associated with exogenous light spectra should also be considered in future studies of coral photosynthesis, as well as the role of photoreceptors in *Symbiodinium* associated with cnidarians living in shallow to deep blue water.
